# Optogenetics meets automated patch clamp

**DOI:** 10.1016/j.isci.2026.116709

**Published:** 2026-07-13

**Authors:** Reetta Penttinen, Ariel Coli, Florian Hintermaier, Nicoletta Murciano, Stephan Holzhauser, Shiqiang Gao, Maria Giustina Rotordam, Michael George, Niels Fertig, Lars Kaestner

**Affiliations:** 1 Nanion Technologies, Munich, Germany; 2 Theoretical Medicine and Biosciences, Medical Faculty, Saarland University, Homburg, Germany; 3 Dynamics of Fluids, Experimental Physics, Saarland University, Saarbrücken, Germany; 4 Heinz Nixdorf Chair of Biomedical Electronics, Technical University of Munich, Munich, Germany; 5 Department of Neurophysiology, Physiological Institute, University of Würzburg, Würzburg, Germany

**Keywords:** channelrhodopsin, automated patch-clamp, NCR1, XXM, stimulating optical lid, SOL

## Abstract

Channelrhodopsins have revolutionized the rapid, contactless modulation of action potentials, driving major advances in both neuroscience and cardiac research. In parallel, the automated patch-clamp (APC) technique has emerged as a powerful platform for drug screening. Here, we present the first biophysical characterization of the Na^+^-selective channelrhodopsin NCR1 2.0 (sharing the rhodopsin part sequences with *Hc*CCR) and the highly Na^+^/Ca^2+^-conductive variant XXM 2.0, using APC combined with integrated optical stimulation. These channels are gaining increasing relevance, as Na^+^- and/or Ca^2+^-dependent signaling plays pivotal roles in cancer biology and the development of anti-cancer therapeutics. The combined application of optogenetics and APC offers a transformative approach to drug screening, opening new avenues for biomedical innovation. This vision also includes further optical manipulations such as uncaging of substances and molecular photoswitches in photopharmacology, which form synergistic approaches with APC.

## Highlights

• Fully integrated device combining automated patch-clamp and optical stimulation
• Biophysical comparison of the Na^+^-conducting channelrhodopsins NCR1 2.0 and XXM 2.0
• NCR1 2.0 is the preferred rhodopsin for applications relying on Na^+^ specificity

## Graphical abstract

**Figure:**
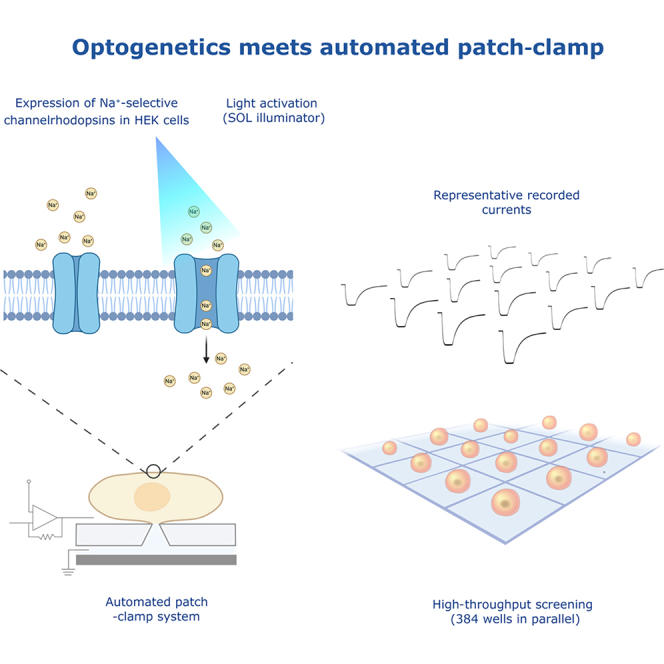

## Teaser

Biological sciences

## Introduction

It was the interaction of light with molecular structures (absorption) that enabled the discovery of cells in the first microscopes.^1^ This presumably contactless observation was a dominating method in cell biology for several centuries. However, with technological progress, the application of light expanded from observation to active manipulation, spreading from mechanical manipulation using laser tweezers^2^ to chemical “switching” of substances by light.^3^ In one of the earliest applications, this switching was a crude destruction of fluorescent dyes leading to the development of a method called “fluorescence redistribution after photobleaching” (FRAP), which led to the discovery of the mobility of lipids in a biological membrane.^4^ The next evolutional step was the invention of the so-called caged compounds, where a biologically active compound is chemically masked, and this mask can be cleaved by light, typically ultraviolet. Examples for caged compounds include caged calcium,^5^ caged InsP_3_,^6^ caged glutamate,^7^ caged ATP,^8^ or caged glutaraldehyde.^9^

**Figure 1:**
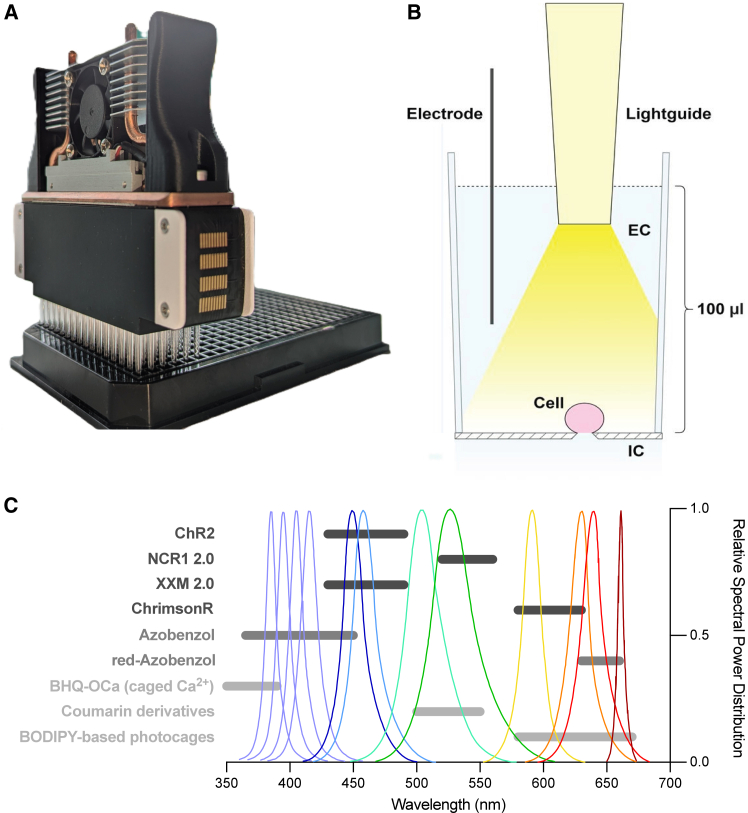
The functionality of the Stimulating Optical Lid (SOL) (A) Example of a SOL illuminator covering one-quarter of the Nanion 384-well chip. One SOL illuminator consists of 96 light guides, one for each well of the plate. A total of four SOL illuminators can be mounted on the plate, covering up to 384 wells. (B) Schematic cross-section of one well of a Nanion 384-well plate with the light guide inside. The panel is illustrative and not drawn to scale. EC and IC refer to extracellular and intracellular solutions, respectively. (C) Available color range for optical stimulation with the SOL. The relative spectral power distribution is plotted against the wavelength of each respective color option. A SOL illuminator contains light-emitting diode boards with one (single color) or two (dual color) spectral specifications, but SOL illuminators for all plotted spectra are available. A selection of light-activated compounds is indicated by gray bars referring to the spectral activation range. A selection of optogenetic tools in terms of channelrhodopsins (including the ones studied in this report) is drawn in dark gray. The most common photoswitches azobenzol and red-azobenzol are plotted in medium gray. Finally, a selection of caged compounds from an example of caged Ca^2+^ (BHQ-OCa) to compounds that are used as cages in several complexes are given in light gray.

With the advent of the genetically encoded fluorescent markers following the discovery of the green fluorescent protein,^10^ the way was paved for the introduction of genetically encoded “switches.” “On” and “off” switches pushed the progress of super-resolution localization methods, such as STORM, PALM, and related methods. With the discovery of channelrhodopsins,^11^ the dream of a direct optical manipulation of electrical properties of cellular membranes became true. However, the simultaneous manipulation and recording of cellular signals required sophisticated setups, complicated procedures, and skilled users.^12^ Here, we show that optogenetic manipulation of ion channels and their recordings can be achieved by an automated process with a fully integrated device (Figure 1). To this end, we directly compared the properties of the Na^+^-channelrhodopsin NCR1 2.0^13^ and the Na^+^/Ca^2+^-channelrhodopsin XXM 2.0.^14^^,^^15^

NCR1 2.0 has 432 amino acids in its full-length form^13^; residues 1–265 were first synthesized and characterized as *Hc*CCR (for *Hyphochytrium catenoides* cation channelrhodopsin, GenBank: OL692497).^16^^,^^17^ NCR1 2.0 comprises residues 11–283 of NCR1 together with several plasma membrane-targeting peptides and has been characterized by markedly enhanced photocurrents.^13^ The *Hc*CCR has been well characterized in terms of the structural foundations of its selectivity^17^ and its atomic structure determined by cryoelectron microscopy.^18^

XXM 2.0 is a variant derived from XXM (the D156H mutant of *Chlamydomonas reinhardtii* ChR2)^19^ with an extra H134Q substitution, addition of membrane-targeting signal peptides, and an N-terminal 11 amino acid truncation.^14^ It is highly selective for Na^+^ and Ca^2+^, whereas its previous application was mainly based on its Ca^2+^ conductance.^14^^,^^15^

Here, we focus on the Na^+^ permeability of NCR1 2.0 and XXM 2.0 and demonstrate the direct optical activation and recording of these engineered channelrhodopsins using a fully automated patch-clamp (APC) platform.

## Results

### Irradiance-dependent photocurrent kinetics

**Figure 2:**
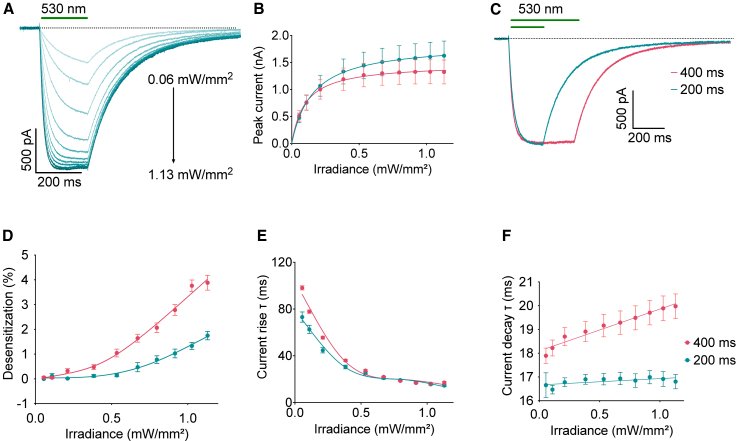
The light intensity dependence of NCR1 2.0 (A) A series of photocurrent traces generated by NCR1 2.0 at −80 mV in response to 200 ms light pulses of incremental irradiance from 0.06 to 1.13 mW/mm^2^, with increasing darkness of the line. The bar on the top shows the duration of illumination. (B) The dependence of the absolute peak current amplitude on the light intensity (irradiance) at two different pulse durations (400 ms, in magenta, and 200 ms, in teal). (C–F) (C) Representative photocurrent traces generated by NCR1 2.0 at −80 mV in response to 0.5 mW/mm^2^ irradiance for a duration of either 400 ms (magenta) or 200 ms (teal). The bars on the top show the duration of illumination. The dependence of degree of desensitization (D), photocurrent rise τ (E), and photocurrent decay τ (F) on the light intensity at two different pulse durations (400 ms, in magenta, and 200 ms, in teal). Desensitization was calculated as the difference between peak current and current at the end of the light illumination divided by the peak current and expressed as a percentage (%). Photocurrent rise and decay τ were calculated using a logistic function. Data are shown as mean ± SEM. 400 ms: *n* = 32 (68); 200 ms: *n* = 27 (50). *n* represents the number of responding cells for a given experimental condition out of the total amount of valid cells given in brackets. The graphs in (F) were fitted by a linear regression. While the slope for the 200 ms light pulses is not significantly different from zero (*p* = 0.3), the slope of the 400 ms values is significantly different from zero (*p* < 0.0001).

To evaluate the light sensitivity and photocurrent kinetics of the channelrhodopsins, HEK293 cells transfected with NCR1 2.0 and XXM 2.0 were illuminated with the respective peak excitation wavelengths at holding potential −80 mV. Increasing irradiances were applied with pulse durations of either 200 or 400 ms to assess the dependence of peak current amplitude, desensitization, and photokinetics on light intensity (Figure 2 for NCR1 2.0; Figure S2 for XXM 2.0).

Representative current traces from NCR1 2.0-transfected cells show that photocurrent amplitude increased with irradiance in a dose-dependent manner until reaching saturation. Peak currents saturated at ∼0.5 mW/mm^2^, independent of pulse durations tested, indicating that channels achieve maximal activation at this irradiance almost immediately and that prolonging the light pulse does not produce a larger peak current.

Photocurrent desensitization, defined as a decline in current amplitude during sustained illumination, was dependent on both irradiance power and pulse duration (Figure 2D). Kinetic analysis revealed that τ rise progressively decreased with increasing irradiance (Figure 2E), while τ decay following light offset remained largely constant across intensities (Figure 2F). Notably, the extent of desensitization in NCR1 2.0 was comparatively modest relative to other channelrhodopsins.^20^^,^^21^

Increasing irradiance produced comparable results to decreasing stimulation order (Figure S3), indicating that stimulus history had no impact on saturation, desensitization or photocurrent kinetics. Repetitive stimulation did not affect the peak current amplitudes of NCR1 2.0 or XXM 2.0 (Figures S4 and S5), indicating that a 10 s pulse interval was sufficient for full recovery of channel activation. Photocurrent kinetics of NCR1 2.0 remained stable during repeated stimulations, whereas XXM 2.0 exhibited slower activation after the initial light stimulation. This change in kinetics of XXM 2.0 suggests incomplete channel recovery during repeated activation, without affecting the maximal current amplitude.

XXM 2.0-transfected cells exhibited a similar light intensity-dependent behavior (Figure S2) to NCR1 2.0. Photocurrents increased with irradiance and saturated at ∼0.9 mW/mm^2^. Desensitization became apparent at irradiances ≥1 mW/mm^2^ and was less pronounced with a 200 ms pulse duration. Current rise and decay exhibited light intensity dependence at irradiances below 1 mW/mm^2^.

For subsequent comparative analyses of photocurrent amplitude and kinetics, stimulation conditions were standardized to 0.5 mW/mm^2^ (200 ms pulses) for NCR1 2.0 and 0.9 mW/mm^2^ (200 ms pulses) for XXM 2.0, as these settings yielded maximal peak currents without inducing desensitization.

### Fluorescence-based evaluation of channelrhodopsin expression and photocurrent correlation

**Figure 3:**
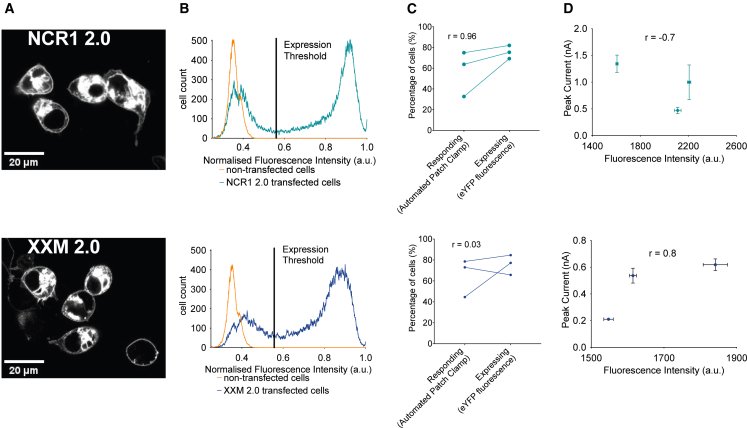
Microscopic imaging and flow cytometry analysis of NCR1 2.0 (top) and XXM 2.0 (bottom) (A) Representative confocal fluorescence images of HEK293 cells transfected with NCR1 2.0 or XXM 2.0, showing subcellular distribution of channelrhodopsins. Scale bars are drawn and annotated within the micrographs. (B–D) (B) Normalized fluorescence intensity distributions for channelrhodopsin-transfected and non-transfected HEK293 cells, with fluorescence expression threshold indicated. For each sample 10,000 events were recorded. The peak of the non-transfected cells was normalized to the maximal count of the transfected cells for graphical purposes. Data are used in (C and D) to determine the threshold of fluorescent cells. (C) Correlation between the percentage of fluorescent cells (flow cytometry measurements) and the percentage of responder cells in APC recordings. Each set of connected data points represents measurements obtained from the same transfection batch. (D) Relation between peak photocurrent at 0.5 and 0.9 mW/mm^2^ for NCR1 2.0 and XXM 2.0, respectively, and mean fluorescence intensity (flow cytometry). Data are shown as mean ± SEM, tested for correlation using the Pearson coefficient. (C and D) Related flow cytometry measurements and APC recordings were always performed simultaneously from the same batch of transfected cells.

In contrast to conventional optogenetic applications, APC does not allow for visual inspection. Therefore, microscopy analysis and flow cytometry were performed separately. The microscopy was used to get general information on the spatial distribution of the channelrhodopsins within the cells, whereas the flow cytometry provided statistical information on the expression level that could be compared with the statistics of the APC measurements. Figure 3A shows the representative images of HEK293 cells transfected with NCR1 2.0 (top) and XXM 2.0 (bottom). Channelrhodopsins are visible in fluorescence microscopy because they are coupled to the fluorescent protein eYFP. Although channelrhodopsins are transmembrane proteins and fluorescence is clearly visible at the cell membrane, a portion of the signal is also observed intracellularly. Flow cytometry was used to assess whether fluorescence intensity, as an indicator of channelrhodopsin expression, correlated with the data obtained from APC.

Figure 3B illustrates the normalized fluorescence intensity distribution obtained from non-transfected and channelrhodopsin-transfected HEK293 cells. The expression threshold used to classify cells as fluorescent is indicated on the plot and was applied to define the population of eYFP-positive (and therefore channelrhodopsin-transfected) cells in subsequent analyses. This classification underlies the quantitative comparison shown in Figure 3C, which contrasts the percentage of expressing cells with the percentage of cells responding to light stimulation as measured by APC. The correlation resulted in Pearson’s r values of +0.96 and + 0.03 for NCR1 2.0 and XXM 2.0, respectively. Both channelrhodopsins were stimulated at their respective maximal excitation wavelengths. Peak current values were obtained using 0.5 mW/mm^2^ for NCR1 2.0 and 0.9 mW/mm^2^ for XXM 2.0. Figure 3D depicts the relation between fluorescence intensity measured by flow cytometry and peak photocurrent recorded using APC, where Pearson’s r values were − 0.7 and + 0.8 for NCR1 2.0 and XXM 2.0, respectively.

### Comparative electrophysiological characterization of NCR1 2.0 and XXM 2.0

**Figure 4:**
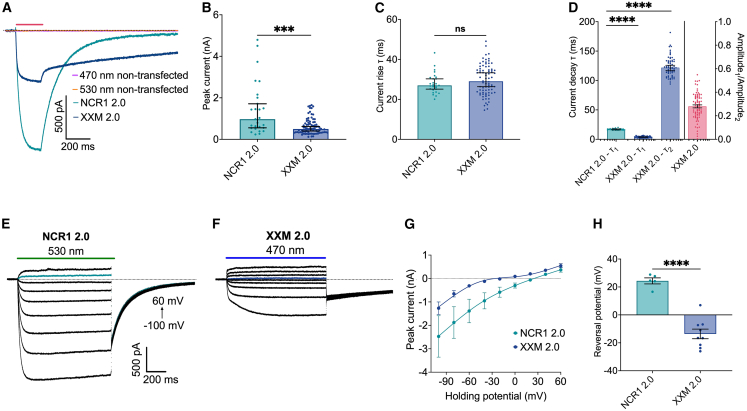
Comparative electrophysiological characterization of NCR1 2.0 and XXM 2.0 (A–C) (A) Representative photocurrent traces recorded from HEK293 cells transfected with NCR1 2.0 (teal) or XXM 2.0 (blue) at −80 mV in response to a 200 ms light pulse (530 nm, 0.5 mW/mm^2^, for NCR1 2.0 and 470 nm; 0.9 mW/mm^2^, for XXM 2.0). Non-transfected HEK293 cells were stimulated using both wavelengths (orange for 470 nm and magenta for 530 nm) as controls. The bar on top shows the duration of illumination. Absolute peak current amplitude (B) and photocurrent rise τ (C) upon light stimulation are shown for both NCR1 2.0 and XXM 2.0. (D–F) (D) Photocurrent decay τ after light offset for NCR1 2.0 and XXM 2.0 is plotted on the left *y* axis and amplitude ratio of the components on the right. Series of photocurrent traces recorded from NCR1 2.0 (E) and XXM 2.0 (F) upon incremental voltage from −100 to +60 mV in 20 mV steps. The bars on the top show the duration of illumination. (G) Current-voltage relationships of NCR1 2.0 and XXM 2.0 are shown as mean ± SEM. (H) The reversal potentials of the current at the end of illumination are shown for both channelrhodopsins. The bars are mean ± SEM, and the dots are data points from individual cells. Statistical significance was assessed using an unpaired *t* test. NCR1 2.0: *n* = 5 (17); XXM 2.0: *n* = 9 (22); ∗∗∗∗ for *p* < 0.0001. *n* represents the number of responding cells for a given experimental condition out of the total amount of valid cells given in brackets. The bars are median and 95% confidence interval (B) or mean ± SEM (C and D); the dots are data points from individual cells. Statistical significance was assessed using a Mann-Whitney test (B), unpaired *t* test (C), and ordinary one-way ANOVA (D). In (B–D), NCR1 2.0: *n* = 28 (51); XXM 2.0: *n* = 82 (115); ∗∗∗∗ for *p* < 0.0001; ∗∗∗ for *p* < 0.001.

To directly compare the photocurrent properties, HEK293 cells expressing NCR1 2.0 or XXM 2.0 were illuminated using 200 ms light pulses of 0.5 mW/mm^2^ (NCR1 2.0) or 0.9 mW/mm^2^ (XXM 2.0), respectively, while held at −80 mV (Figures 4A–4D). Non-transfected cells were recorded in parallel and stimulated with both wavelengths as controls. Reversal potentials were determined using a voltage-step protocol from −100 mV to +60 mV in 20 mV increments, with 1 s light pulses of 0.5 mW/mm^2^ (NCR1 2.0) or 0.9 mW/mm^2^ (XXM 2.0) applied at each potential (Figures 4E–4H). NCR1 2.0 generated larger inward photocurrents compared to XXM 2.0 (Figures 4A and 4B) at −80 mV. Kinetic comparison showed no difference in τ rise between the channelrhodopsins (Figure 4C), indicating similar activation kinetics. However, deactivation kinetics diverged distinctly. NCR1 2.0 displayed a fast, mono-exponential current decay, while XXM 2.0 exhibited substantially slower double-exponential deactivation kinetics (Figure 4D).

Voltage-step analysis revealed distinct current-voltage relationships (Figures 4E and 4F). NCR1 2.0 photocurrents reversed at +24.4 mV, whereas XXM 2.0 reversed at −13.6 mV (Figures 4G and 4H), indicating a stronger sodium selectivity for NCR1 2.0. Replacing intracellular K^+^ with Na^+^ caused a pronounced negative shift in the reversal potential of NCR1 2.0, whereas the corresponding shift for XXM 2.0 was markedly smaller (Figure S6). This difference is consistent with a higher Na^+^ selectivity of NCR1 2.0, while XXM 2.0 may exhibit broader cation permeability, including K^+^.

## Discussion

Historically, the functional characterization of channelrhodopsins has relied on manual patch-clamp recordings, which provided detailed but low-throughput data, limiting systematic comparisons across constructs. The integration of APC platforms with optical stimulation has transformed this workflow. APC enables parallel, unbiased whole-cell recordings with controlled light delivery, increasing throughput and reproducibility, while maintaining high-resolution kinetic measurements. This approach allows the systematic assessment of intensity dependence and channel properties, positioning APC as a powerful method for channelrhodopsin characterization.^16^^,^^22^^,^^23^ Here, we compare the biophysical properties of NCR1 2.0 and XXM 2.0 using high-throughput APC and integrated optical stimulation. In the future, this approach could be applied to other light-activated channels and to studies employing photoswitchable or caged compounds, building upon previous photopharmacological applications of high-throughput APC systems.^24^

The SyncroPatch 384 system equipped with stimulating optical lid (SOL) was utilized to perform the first direct systematic comparison of NCR1 2.0^13^ and XXM 2.0.^15^

Fluorescence microscopy confirmed successful transfection with a visible membrane-localized signal, while partial intracellular fluorescence, most likely in the endoplasmic reticulum and the Golgi apparatus, indicated that not all expressed protein had trafficked to the plasma membrane. Flow cytometry data showed a correlation with the fraction of cells responding to light in APC, although the response rate detected by APC was slightly lower than that measured by flow cytometry.

Peak photocurrent amplitude and fluorescence intensity showed a divergent situation: NCR1 2.0 exhibited a negative Pearson’s r, while XXM 2.0 showed a positive one. We do not intend to overinterpret these Pearson r values based on 3 data points each. APC measures photocurrents only from the plasma membrane-localized channelrhodopsins, whereas flow cytometry quantifies the total cellular fluorescence, including intracellular pools retained in the secretory pathway or other endomembranes. Therefore, high fluorescence does not necessarily translate into high photocurrent if a substantial fraction of the protein fails to reach the plasma membrane, a common limitation for channelrhopdopsins.^25^ In this context, the negative Pearson’s r observed for NCR1 2.0 may be consistent with reduced membrane trafficking efficiency at higher total expression levels, for instance, due to saturation of trafficking/processing capacity and/or increased intracellular retention or aggregation. So, comparison of the fluorescence intensity and photocurrent amplitude revealed no clear positive correlation, consistent with the heterogeneous subcellular localization of expressed channelrhodopsin. Consequently, cells with high fluorescence may not necessarily exhibit large photocurrents if a substantial fraction of the protein remains intracellular. This is a general challenge for the expression of membrane-targeted proteins and was also reported for genetically encoded biosensors such as membrane potential sensors.^26^ Additionally, further confounding factors such as protein aggregation or membrane saturation may alter the relation between fluorescence detection and photocurrent. Consistent with expectations, non-transfected controls displayed no light-induced currents, confirming that the observed photocurrents recorded with APC originated from the channelrhodopsins.

Channelrhodopsin desensitization is a common feature of most known channelrhodopsins and a generally undesirable characteristic, as it limits the duration and stability of light-evoked responses.^27^ The desensitization generally reflects light-driven accumulation of channels in long-lived non-conducting photocycle states, such that the steady-state current declines even though illumination continues; in channelrhodopsin-2, prolonged illumination can populate secondary photocycles originating from late intermediates that promote functional inactivation.^28^ In some rhodopsins, this can be further exacerbated by activity-dependent intracellular acidification linked to proton permeation, which can reduce current amplitude during sustained stimulation.^29^ Increased irradiance does not further increase peak current amplitudes once channel opening is saturated, but instead accelerates the population of these inactive states, thereby enhancing desensitization; structural and spectroscopic studies have shown that desensitized states are associated with distinct retinal configurations that stabilize non-conducting conformations.^20^^,^^30^ This principle is well illustrated by RubyACR, where sequential absorption of multiple photons drives channels into a bistable, long-lived non-conducting state, causing pronounced current loss during sustained illumination.^21^ Both NCR1 2.0 and XXM 2.0 produced photocurrents that showed similar desensitization with increased irradiance, but at a relatively low level compared with other rhodopsins.^21^^,^^31^^,^^32^ Such minimal desensitization indicates that these channels reach maximal open probability at moderate irradiance and do not strongly accumulate in desensitized states, resulting in stable peak currents and reproducible responses during repeated or prolonged stimulation.

Direct electrophysiological comparison revealed distinct functional differences between the two channelrhodopsins. Both channelrhodopsins displayed a τ rise below 40 ms, confirming rapid response to light stimuli. NCR1 2.0 exhibited larger inward currents, fast monoexponential deactivation, a more positive reversal potential (+24.4 mV), and a pronounced negative shift in reversal potential upon replacement of the intracellular K^+^ with Na^+^. Together, these properties are consistent with a comparatively higher Na^+^ selectivity. These results are in rough agreement with the data obtained from *Hc*CCR,^16^ which shares the rhodopsin part sequences with NCR1 2.0. Its profile makes NCR1 2.0 a promising candidate for applications requiring strong and precise sodium influx, as outlined in a recent review.^33^ In contrast, XXM 2.0 displayed smaller currents, double-exponential deactivation, a more negative reversal potential (−13.6 mV), and a less-pronounced shift in reversal potential when replacing intracellular K^+^ with Na^+^, suggesting lower Na^+^ selectivity, i.e., an additional decent Ca^2+^ conductance as previously reported.^14^^,^^15^ The double-exponential deactivation kinetics of XXM 2.0 indicates the presence of two kinetically distinct open states. A fast component reflects rapid channel closure immediately after light offset, whereas a slower component likely corresponds to a stabilized conductive state that decays more slowly. Such multi-component closing kinetics are well described for channelrhodopsins and arise from differences in their photocycles and structural determinants that stabilize the open conformation.^34^^,^^35^ The slower deactivation of XXM 2.0 compared to NCR1 2.0 can be attributed to its distinct molecular design and ion selectivity profile. XXM 2.0 was engineered to enhance Ca^2+^ permeability, and structural modifications that increase divalent cation conductance are known to prolong the lifetime of the open state, resulting in slower channel closure.^36^^,^^37^^,^^38^ In contrast, the Na^+^-selective NCR1 2.0 follows a faster, predominantly mono-exponential deactivation, due to its quite different pore architecture and gating energetics. Such characteristics may benefit applications requiring sustained depolarization but reduce temporal precision.

Methodologically, APC provides several key advantages for optogenetic studies. Synchronized illumination enhances statistical reliability and reproducibility and enables precise comparison of stimulation parameters, channel properties, and cell populations in parallel. The integration of programmable light delivery ensures consistent irradiance control, yielding results comparable to manual patch-clamp.^16^ Collectively, these features position APC as a reliable, high-throughput platform for detailed channelrhodopsin characterization.

Beyond its technical and methodological strengths, APC can also facilitate the translation of optogenetic tool development into biologically and medically relevant applications. An example is the use of Na^+^-channelrhodopsins in genetically modified and *in vitro*-grown erythrocytes^39^ following the concept of utilizing these erythrocytes as drug carriers that can upon light stimulation release their cargo.^33^

Furthermore, by enabling rapid and precise functional screening, APC supports the identification and characterization of channelrhodopsins engineered for specific applications, such as probing how sodium influx influences invadopodia formation and cancer cell proliferation.^33^

Invadopodia are specialized protrusions formed by invasive cancer cells that facilitate tissue infiltration, degradation of the extracellular matrix (ECM), and cellular invasion, all of which are key steps in metastasis formation. These structures are enzymatically active and contribute to local acidification, weakening ECM integrity, and promoting cancer cell migration.^40^^,^^41^ Recent studies have revealed a strong connection between sodium signaling and invadopodia function: voltage-gated Na^+^ channels are frequently upregulated in metastatic cancer cells and increased expression correlates with enhanced motility, invasiveness, and ECM remodeling. These findings suggest that Na^+^ influx plays a pivotal role in cancer progression.^42^^,^^43^

In this context, optogenetic tools like Na^+^-channelrhodopsins offer a powerful means to manipulate Na^+^ dynamics with high temporal precision. Although invadopodia cannot be directly evaluated by APC, APC-based characterization and screening enable the identification of channelrhodopsins with optimized properties. This allows researchers to use the most suitable variants to investigate Na^+^-dependent processes in cancer cells. Such an approach could further elucidate the relationship between Na^+^ signaling and cancer cell behavior, ultimately contributing to a deeper understanding of cancer progression and the development of therapeutic strategies. Given the established link between Na^+^ signaling and metastatic activity, optogenetic modulation of Na^+^ channels provides a unique tool to dissect the electrophysiological basis of cancer cell invasiveness.

In summary, NCR1 2.0 combines high current amplitude, strong Na^+^ selectivity, and rapid kinetics, making it the more favorable channelrhodopsin for precise Na^+^-based applications. XXM 2.0, with smaller currents and slower deactivation, may be more suitable where prolonged depolarization is desired. These findings highlight how APC accelerates channelrhodopsin characterization, enables systematic variant screening, and supports rational engineering of optogenetic tools for targeted applications, such as better understanding the role of Na^+^ in cancer cell migration.

The APC in combination with SOL opens new avenues even beyond the field of optogenetics. This includes the field of photolytic uncaging^3^ of signaling molecules, such as calcium, ATP, or glutamate, as well as reversible photo-switching of drugs.^44^ This study demonstrates that APC combined with optical stimulation constitutes a robust, high-throughput platform for characterizing optogenetic actuators. Beyond optogenetics, this integration paves the way for approaches in photopharmacology and optical compound uncaging.

### Limitations of the study

Several factors constrain the interpretation of this work. First, the experiments rely exclusively on HEK293-overexpression systems, which may not fully reproduce the trafficking efficiency, membrane composition, or ion-handling environment of native cells, potentially affecting the behavior of both channelrhodopsins and their photocurrents. Second, APC, while high-throughput, imposes strict solution conditions and lacks real-time visual confirmation of membrane localization, meaning that intracellularly retained proteins — clearly visible in the microscopy data — cannot be distinguished from functional membrane-inserted channels during electrophysiological recordings. Third, the correlation analyses between fluorescence and photocurrent are based on a small number of transfection batches, limiting the robustness of conclusions regarding expression-function relationships. Additionally, the study focuses on sodium permeability under a specific set of ionic conditions, leaving the broader ion-selectivity profiles and physiological performance of NCR1 2.0 and XXM 2.0 under more complex or dynamic environments unexplored. Finally, while APC provides precise irradiance control, the optical stimulation geometry differs from typical microscopy-based optogenetics, which may influence activation kinetics and desensitization behavior, and thus should be considered when extrapolating these findings to *in vivo* or cell-type-specific applications.

## Resource availability

### Lead contact

Requests for further information and resources should be directed to and will be fulfilled by the lead contact, Lars Kaestner (lars_kaestner@me.com).

### Materials availability

This study did not generate new unique reagents.

### Data and code availability

• All data reported in this paper will be shared by the lead contact upon request.
• This paper does not report original code.
• Any additional information required to reanalyze the data reported in this paper is available from the lead contact upon request.

## Acknowledgments

This research was funded by the European Community in the Marie Skłodowska-Curie project no. 101120168—INNOVATION.

## Author contributions

Conceptualization, N.M., S.H., M.G.R., and L.K.; methodology, R.P., A.C., F.H., N.M., S.H., S.G., M.G.R., M.G., and N.F.; investigation, R.P., A.C., and F.H.; writing – original draft, R.P., A.C., N.M, M.G.R., and L.K.; writing – review and editing, F.H., S.H., S.G., M.G., and N.F.; funding acquisition, M.G.R., N.F., and L.K.; resources, S.G., M.G., N.F., and L.K.; supervision, N.M., S.H., M.G.R., N.F., and L.K.

## Declaration of interests

R.P., N.M., S.H., M.G.R., M.G., and N.F. are employees of Nanion Technologies GmbH, the manufacturer of the device presented in Figure 1 and used for data acquisition of Figures 2 and 4 and partly of Figure 3. L.K. is a consultant for Cysmic GmbH, which has no relation to this manuscript.

## Declaration of generative AI and AI-assisted technologies in the writing process

During the preparation of this work, the authors used Copilot (Microsoft Corporation) for refining the language (none of the authors is a native English speaker). After using this tool or service, the authors reviewed and edited the content as needed and take full responsibility for the content of the publication.

## STAR★Methods

### Key resources table

**Table:** 

REAGENT or RESOURCE	SOURCE	IDENTIFIER
**Chemicals, peptides, and recombinant proteins**		

TryPLE	Thermo Fisher Scientific	Cat. # 12605036
Hanks’ Balanced Salt Solution	PAN-Biotech	Cat. #P04-34100
jetPEI® HTS DNA transfection reagent	Polyplus-transfection (distributed by Avantor/VWR)	Cat. # 101000053
Opti-MEM Reduced Serum Medium	Thermo Fisher Scientific	Cat. # 11564506
Gibco™ DMEM, high glucose	Thermo Fisher Scientific	Cat. # 11574486
Fetal bovine serum (FBS), standard, South America origin, 0.2 μm sterile filtered	PAN-Biotech	Cat. No. P30-3306
Penicillin–Streptomycin (Pen/Strep)	Sigma-Aldrich	Cat. #P0781
Phosphate-buffered saline (PBS)	Thermo Fisher Scientific	Cat. # 10010023

**Experimental models: Cell lines**		

HEK293	Leibniz Institute DSMZ–German Collection of Microorganisms and Cell Cultures	ACC305

**Recombinant DNA**		

pcDNA3.1-NCR12.0-eYFP	this paper	available upon request
pcDNA3.1-XXM2.0-eYFP	this paper	available upon request

**Software and algorithms**		

PatchControl384 v3.2.2	Nanion Technologies GmbH	https://www.nanion.de/
DataControl384 v3.6.0	Nanion Technologies GmbH	https://www.nanion.de/
GraphPad Prism v10	GraphPad Software	https://www.graphpad.com/
LAS X v4.9.0.	Leica Microsystems	https://www.leica-microsystems.com/products/microscope-software/p/leica-las-x-ls/

**Other**		

SyncroPatch 384 SOL	Nanion Technologies GmbH	Cat. # 21 3231
NPC-384T S-type chips	Nanion Technologies GmbH	Cat. # 22 2101
Internal KF	Nanion Technologies GmbH	Cat. # 08 3007
External Standard	Nanion Technologies GmbH	Cat. # 08 3001

### Experimental model and study participant details

Human embryonic kidney 293 (HEK293) cells were used in this study. HEK293 cells (ACC 305) were obtained from the Leibniz Institute DSMZ–German Collection of Microorganisms and Cell Cultures (Braunschweig, Germany). The HEK293 cell line is of human origin and is derived from embryonic kidney tissue transformed with adenovirus type 5 DNA. It is classified as risk category 1 according to the German Central Commission for Biological Safety (ZKBS). HEK293 cell line identity was authenticated by the provider (Leibniz Institute DSMZ) using short tandem repeat (STR) analysis. HEK293 cells were routinely tested and confirmed to be free of mycoplasma contamination.

HEK293 cells were maintained under standard culture conditions as described in the method details section and were transiently transfected with the Na^+^-channelrhodopsin NCR1 2.0^13^ and the Na^+^/Ca^2+^-channelrhodopsin XXM 2.0^14^^,^^15^ using the jetPEI HTS DNA transfection reagent (Cat. # 101000053 Polyplus-transfection/Sartorius distributed by VWR International GmbH, Darmstadt, Germany). Experiments were conducted using low-passage cells (passages 9–12).

### Method details

#### Cell culture and harvesting

Human embryonic kidney (HEK) 293 cells (ACC 305, Leibniz Institute, Braunschweig, Germany) were maintained in high-glucose Dulbecco’s Modified Eagle’s Medium (DMEM; Cat. # 11574486, Gibco, Thermo Fisher Scientific, Darmstadt, Germany) supplemented with 10% fetal bovine serum (FBS; Cat. #P30-3306 PAN-Biotech GmbH, Aidenbach, Germany) and 1% penicillin-streptomycin (Cat. #P0781, Sigma-Aldrich Chemie GmbH, Steinheim am Albuch, Germany). Cells were cultured in a humidified incubator at 37°C with 5% CO_2_. Cells were routinely passaged and used for experiments between passages 9 and 12. For each experiment, cells were seeded into T175 culture flasks (Thermo Fisher Scientific, Darmstadt, Germany) or 6-well culture plates (Greiner Bio-One GmbH, Frickenhausen, Germany) at a density sufficient to achieve confluency on the desired day. The culture medium was refreshed every 1–2 days, and the cells were monitored to ensure they reached 70–80% confluency before transfection.

Transfection was carried out using the JetPEI HTS DNA transfection reagent (Cat. # 101000053, Polyplus-transfection/Sartorius distributed by VWR International GmbH, Darmstadt, Germany), according to the manufacturer’s instructions. For the transfection, 83 μL or 4.5 μL of plasmid DNA (1 μg/μL) were used to a T175 flask or a 6-well plate respectively, to achieve a final DNA-to-surface-area ratio of 4.5 μg per 9.5 cm^2^. DNA was then mixed with JetPEI reagent in Opti-MEM Reduced Serum Medium (Cat. # 11564506, Gibco, Thermo Fisher Scientific, Darmstadt, Germany) and incubated for 20–25 min at room temperature to allow for DNA–reagent complex formation. The transfection mix was then added dropwise to the cells, which had been seeded 1–2 days prior to transfection, so as to have a confluency of approximately 70–80%. After the addition of the transfection mix, the cells were incubated at 37°C for an additional 48–72 h. On the day of the experiments, cells were harvested following Nanion’s standard procedure.^45^ In brief, cells were washed twice with HBSS (Hank’s Balanced Salt Solution, Cat. #P04-34100, PAN-Biotech GmbH, Aidenbach, Germany) and then detached with TrypLE™ (1×, Gibco, Cat. # 12605036, Thermo Fisher Scientific, Darmstadt, Germany) by 10 min incubation at 37°C. Cells were resuspended in 4°C External Standard solution (for composition, see below) and incubated at 4°C for 15 min. The final suspension was then transferred to a cell hotel set at 10°C with orbital shaking.^45^

#### Flow cytometer analysis

Flow cytometer analysis was performed using CyFlow Cube 6 flow cytometer (Sysmex GmbH, Norderstedt, Germany) in parallel to patch clamp recordings. After the enzymatic dissociation, 100 μL of cell suspension were taken and added to 1 mL of External Standard solution (for composition, see below). The suspension was then transferred to 2 mL round-bottom test tubes (Sysmex GmbH Norderstedt, Germany) and resuspended before analysis. Flow cytometry data shown in Figures 3B–3D represent analysis of 10,000 cells per sample, performed using the CyFlow Cube 6 flow cytometer’s software, Excel (Office 365, Microsoft Corporation, Redmond, WA, USA) and Prism 10 (v10, GraphPad software, Boston, MA, USA).

#### Imaging

For live-cell imaging, HEK293 cells were seeded and transfected directly in standard 6-well culture plates (Greiner Bio-One GmbH, Frickenhausen, Germany). At 24–48 h post-transfection, the culture medium was gently aspirated, and cells were carefully washed once with phosphate-buffered saline (Gibco Phosphate Buffered Saline (PBS), pH 7.4 (1×), Cat. # 10010023, Thermo Fisher Scientific, Darmstadt, Germany) pre-warmed at 37°C, to remove residual debris while minimising cell disturbance. Fresh 37°C DMEM was then added to each well. Imaging was performed directly in the culture plates using a Leica Stellaris 5 microscope (Leica Microsystems, Mannheim, Germany) equipped with an HC PL APO CS2 63×/1.40 oil objective (Leica Microsystems, Mannheim, Germany). Image acquisition was performed using Leica LAS X software while processing was done using Leica LAS X software and Fiji (ImageJ, release 2.16.0, National Institutes of Health, Bethesda, MD, USA).

#### Automated patch-clamp recordings

Whole-cell automated patch-clamp (APC) recordings were performed 48–72 h after transfection using the SyncroPatch 384 (Nanion Technologies GmbH, Munich, Germany). Experiments were conducted at room temperature using NPC-384T S-type chips (Cat. # 222101, Nanion Technologies GmbH, Munich, Germany) with one hole per well (resistance 2.4–3.8 MΩ). The recording solutions contained (in mM) 110 KF, 10 KCl, 10 NaCl, 10 EGTA, and 10 HEPES, pH 7.2 adjusted with KOH (Internal KF110, Cat. # 08 3007, Nanion Technologies GmbH, Munich, Germany), and 140 NaCl, 4 KCl, 2 CaCl_2_, 1 MgCl_2_, 5 D-glucose monohydrate, 10 HEPES, pH 7.4 adjusted with NaOH (External Standard, Cat. # 08 3001, Nanion Technologies GmbH, Munich, Germany). Data were acquired using PatchControl384 v3.2.2 (Nanion Technologies GmbH, Munich, Germany), sampled at 20 kHz.

Photostimulation was provided by LUXEON Z Color Line light-emitting diodes (LEDs, Lumileds, Schiphol, The Netherlands), either LXZ1-PB01 (470 ± 10 nm, 38 lm at 500 mA | 25°C) or LXZ1-PM01 (530 ± 10 nm, 118 lm at 500 mA | 25°C). Each LED type was arranged as one module, respectively with a 6 × 16 matrix that covered a quarter of the 384-well plate (Figure 1A). Depending on the recording, either one or both LED modules were used. A custom-designed light guide (Zeonex 350 R, light transmittance 92%, Zeon, Tokyo, Japan) was attached to each LED to direct illumination closer to the cell (Figure 1B). The LEDs were driven by the SyncroPatch 384 SOL (Cat. # 21 3231, Nanion Technologies GmbH, Munich, Germany) and controlled by the accompanying software (Nanion Technologies GmbH, Munich, Germany). The available color range for optical stimulation with the SOL is presented in Figure 1C, and the parameter range for optimal stimulation is presented in Table S1.

HEK293 cells transfected with NCR1 2.0 or XXM 2.0 were stimulated at their respective peak excitation wavelengths (530 nm for NCR1 2.0; 470 nm for XXM 2.0). Non-transfected HEK293 cells were stimulated using both wavelengths. Separate sets of wells on the NPC-384T chip were assigned to channelrhodopsin-expressing and non-transfected cells, enabling parallel recordings. To characterize light-dependence, cells were held at −80 mV and exposed to increasing irradiances (0.06–1.13 mW/mm^2^ for NCR1 2.0; 0.10–1.96 mW/mm^2^ for XXM 2.0) for 200 ms or 400 ms light pulses at 10 s intervals. To test whether stimulus order or repetitive light application affected responses, descending irradiance sequences (1.13–0.06 mW/mm^2^) and seven repetitive pulses at 0.5 mW/mm^2^ (200 ms duration) were also applied to NCR1 2.0-transfected cells. The irradiance–LED current relationship is shown in Figure S1. Reversal potentials were estimated using a voltage step protocol from −100 mV to +60 mV (20 mV increments, 20 s interval between light pulses) with a holding potential of −80 mV and 1 s light pulses (0.5 mW/mm^2^ for NCR1 2.0; 0.9 mW/mm^2^ for XXM 2.0).

Cells with seal resistance >200 MΩ and a membrane capacitance >4 pF were considered as total cells. Cells with peak photocurrents <−100 pA at maximum light intensity or most negative voltage (only for reversal potential analysis) were classified as responders.

### Quantification and statistical analysis

Photocurrent traces were analyzed using DataControl384 v3.6.0 (Nanion Technologies GmbH, Munich, Germany). Current rise for both NCR1 2.0 and XXM 2.0 and decay kinetics for NCR1 2.0 were fitted using a mono exponential logistic function. Decay kinetics for XXM 2.0 were fitted using double exponential logistic function. Desensitisation was calculated as the difference between peak current and current at the end of the light illumination, divided by the peak current and expressed as a percentage (%). Peak current for reversal potential analysis was measured as extremum at the end of light stimulation. Statistical analysis was performed using Prism 10 (v10, GraphPad software, Boston, MA, USA). Data are presented as mean ± SEM or as median with 95% confidence intervals, as indicated in figure legends. Individual cell data points are shown where appropriate. Curves were fitted using spline interpolation for photokinetic and desensitization analyses and for XXM 2.0 peak current amplitude. Michaelis–Menten fitting was used for NCR1 2.0 peak current amplitude.

Normality was assessed via Kolmogorov-Smirnov test. For normally distributed data, unpaired t-tests and ordinary one-way ANOVA tests were used; otherwise, Mann–Whitney tests were applied. n represents the number of responding cells out of the total amount of valid cells recorded.
