# Supplementary material for: Optogenetics meets automated patch clamp
Source: iScience. 2026 Jul 13;29(8):116709. doi: 10.1016/j.isci.2026.116709 (PMC13382327; doi:10.1016/j.isci.2026.116709)
Supplement: Document S1. Figures S1–S6 and Table S1 [file mmc1.pdf]

iScience, Volume *29*

Supplemental information

Optogenetics meets automated patch clamp

Reetta Penttinen, Ariel Coli, Florian Hintermaier, Nicoletta Murciano, Stephan Holzhauser, Shiqiang Gao, Maria Giustina Rotordam, Michael George, Niels Fertig, and Lars Kaestner

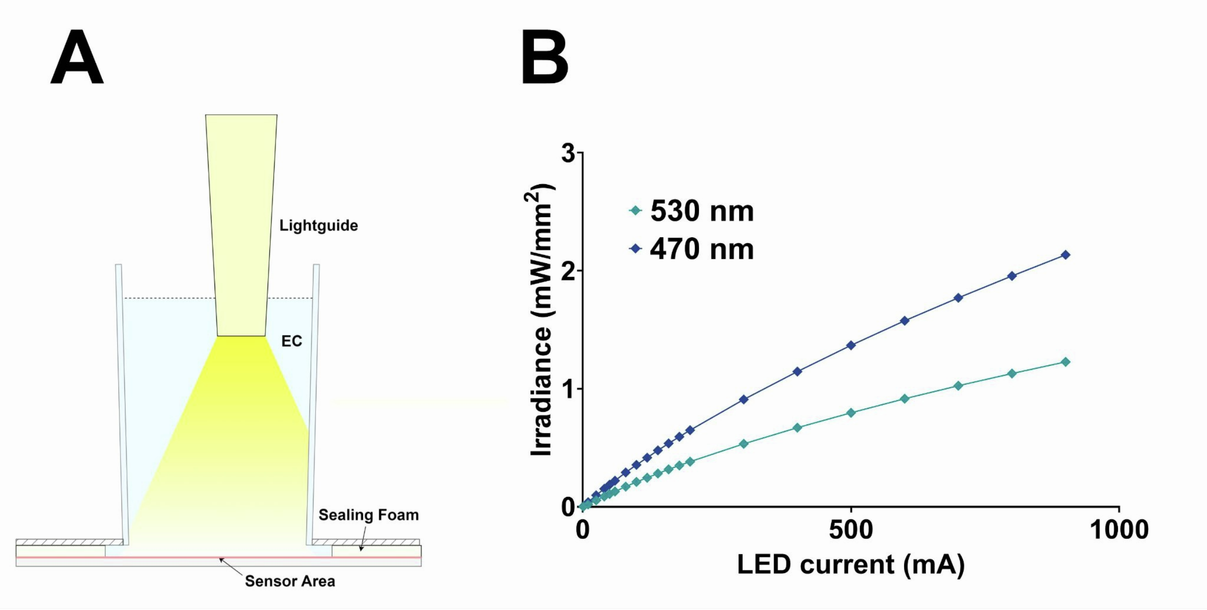

Figure S1: *Measurement of irradiance values for SOL. (A) Experimental setup used for irradiance measurements. Wattage was recorded using a S170C sensor from Thorlabs (Newton, New Jersey, USA) and data acquisition was performed with a PM400 power meter (Thorlabs, Newton, New Jersey, USA). The irradiance was calculated by dividing wattage through the well area. The schematic shown is not to scale. (B) Irradiance shown as a function of LED current for the two different wavelengths. LEDs were pulsed for 100 ms at increasing current levels, and the mean power during the 100 ms pulse was used to calculate the irradiance.*

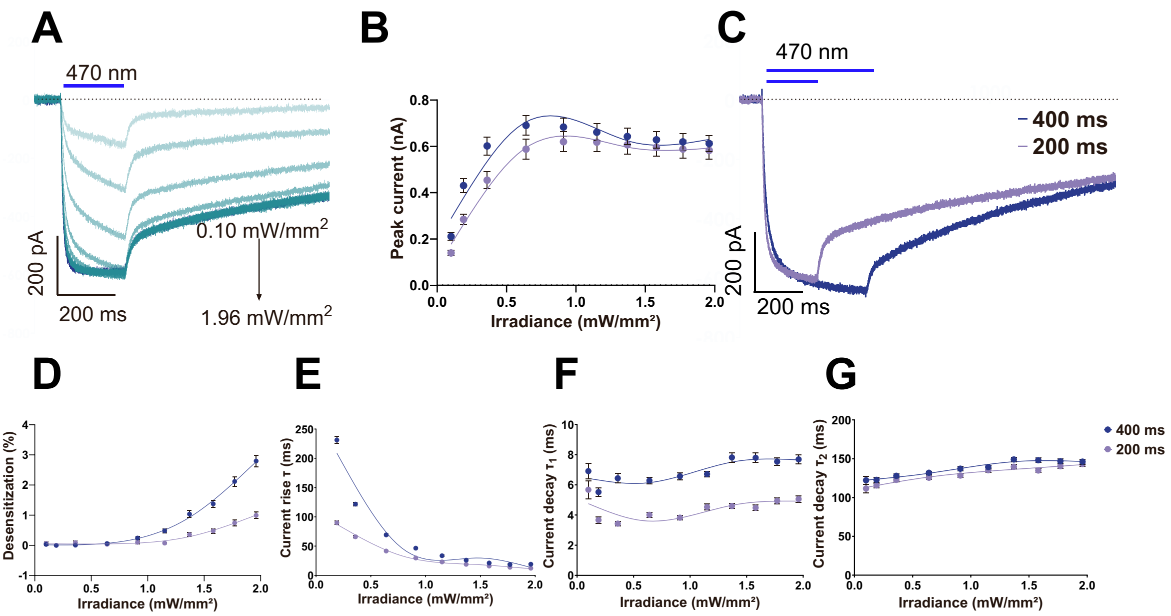

Figure S2: *The light intensity dependence of XXM 2.0. (A) A series of photocurrent traces generated by XXM 2.0 at −80 mV in response to 200 ms light pulses of incremental irradiance from 0.10 mW/mm² to 1.96 mW/mm², with increasing darkness of the line. The bar on top shows the duration of illumination. (B) The dependence of the absolute peak current amplitude on the light intensity (irradiance) at two different pulse durations (400 ms, in blue and 200 ms, in violet). (C) Representative photocurrent traces generated by XXM 2.0 at −80 mV in response to 0.9 mW/mm² irradiance for a duration of either 400 ms or 200 ms. The bars on top show the duration of illumination. The dependence of degree of desensitization (D), photocurrent rise τ (E), and photocurrent decay components $τ_1$ (F) and $τ_2$ on the light intensity at two different pulse durations (400 ms, in blue and 200 ms, in violet). Photocurrent rise and decay τ were calculated with mono (rise) and double (decay) exponential logistic functions. Data are shown as mean ± SEM. 400 ms: n = 89(138); 200 ms: n = 82(115). n represents the number of cells for a given experimental condition out of the total amount of valid cells given in brackets.*

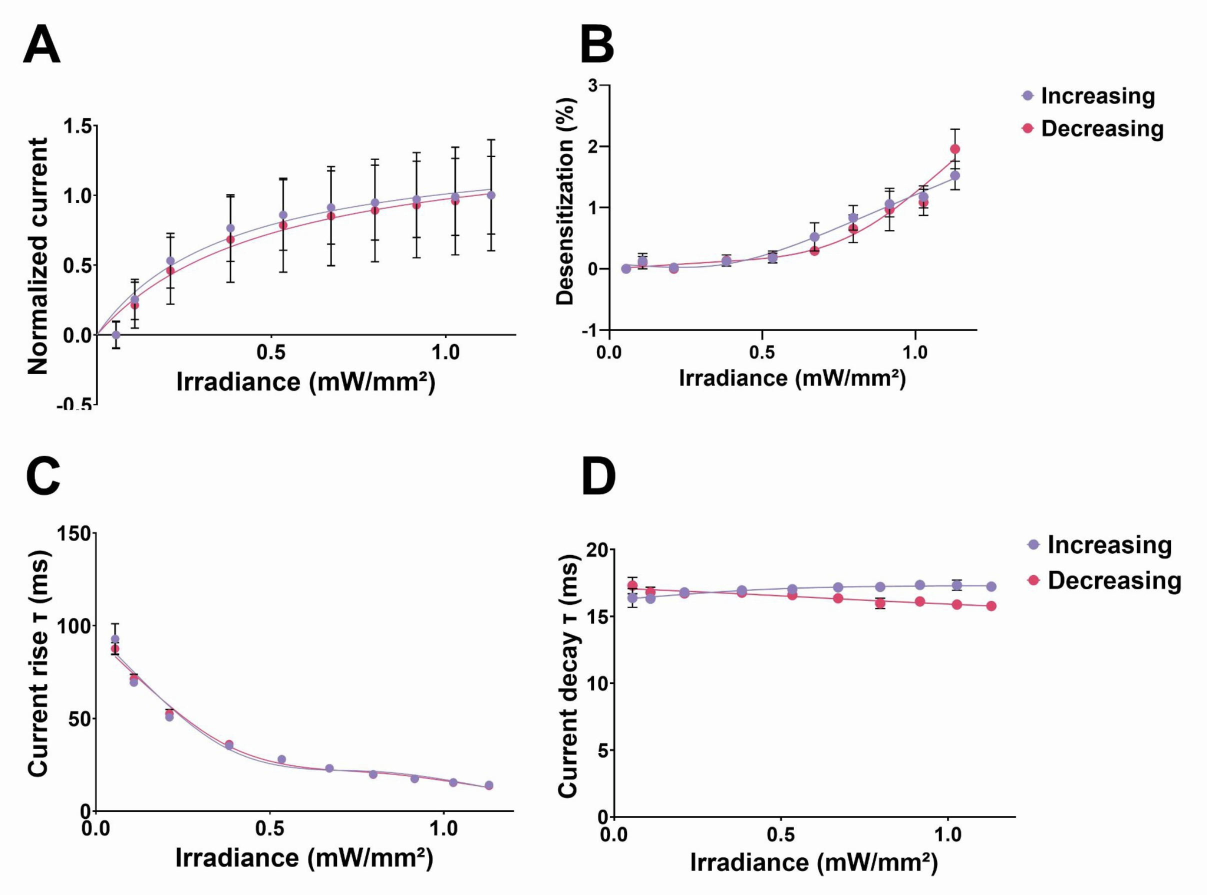

***Figure S3**: Order-dependent effect of light stimulation. Normalized peak current amplitude (A), desensitization (B), current rise τ (C), and current decay τ (D) of NCR1 2.0 measured upon variation of the 200 ms irradiance from low to high and from high to low values. Data are shown as values of individual cells with mean ± SEM. Increasing: n = 19(31); decreasing: n = 8(19). n represents the number of cells for a given experimental condition out of the total amount of valid cells given in brackets.*

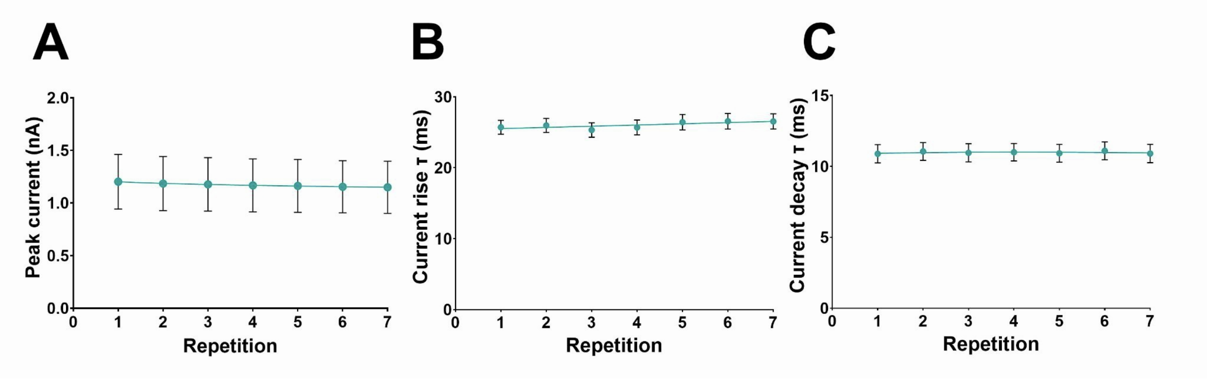

Figure S4*: The effect of multiple light stimulations on peak current amplitude (A), current rise τ (B) and current decay τ (C) of NCR1 2.0. Seven repetitive 200 ms light pulses of irradiance of 0.5 mW/mm² were used to stimulate NCR1 2.0. Data are shown as values of individual cells with mean ± SEM. n = 22(38). n represents the number of cells for a given experimental condition out of the total amount of valid cells given in brackets.*

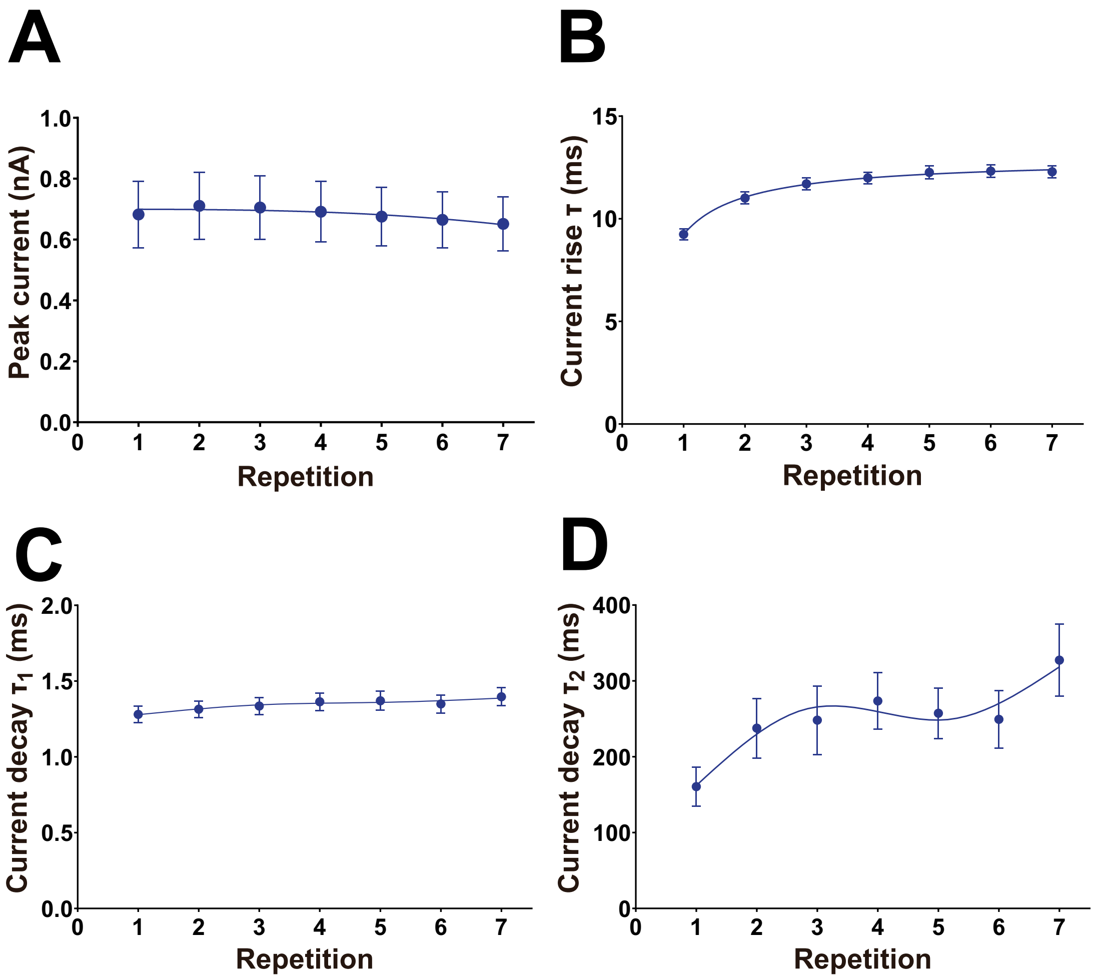

Figure S5: *The effect of multiple light stimulations on peak current amplitude (A), current rise τ (B), current decay* τ_1 *(C) and current decay* τ_2 *(D) of XXM 2.0. Seven repetitive 200 ms light pulses of irradiance of 0.9 mW/mm² were used to stimulate XXM 2.0. Data are shown as values of individual cells with mean ± SEM. n = 28(41). n represents the number of cells for a given experimental condition out of the total amount of valid cells given in brackets.*

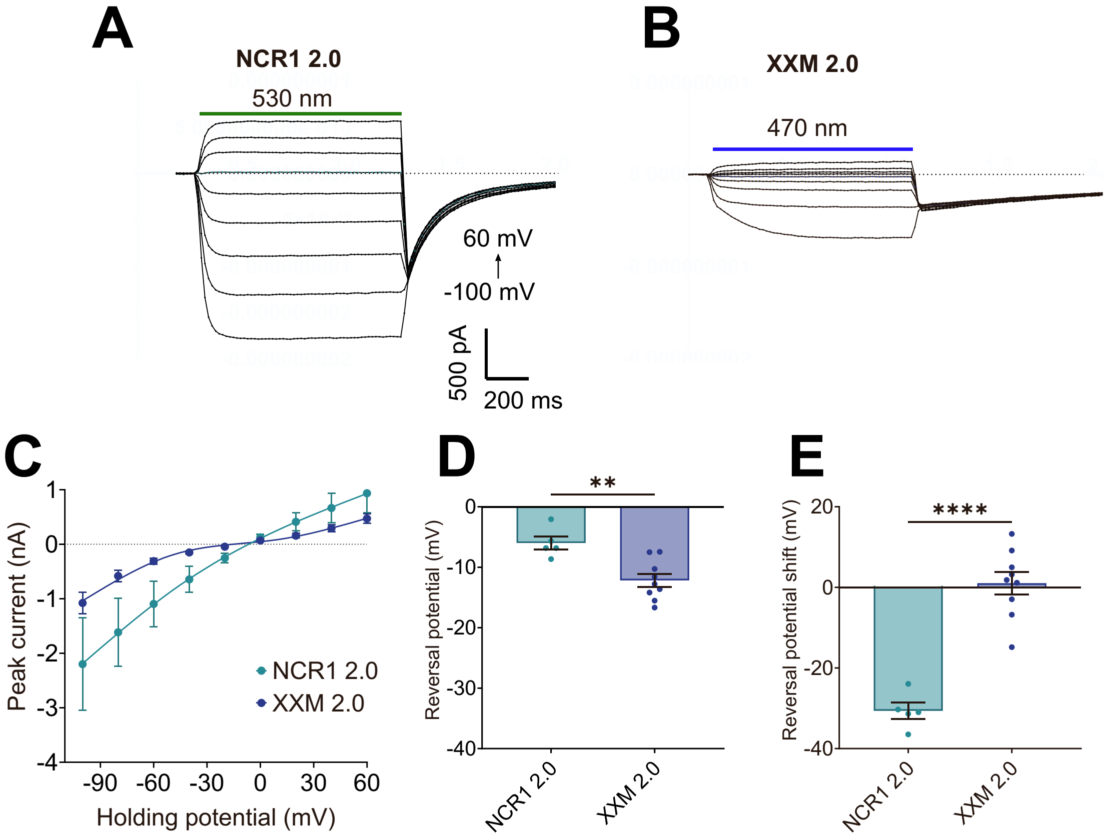

Figure S6**: The reversal potential shifts after replacement of intracellular K^+ to Na^+. Series of photocurrent traces recorded from NCR1 2.0 (A) and XXM 2.0 (B) upon incremental voltage from −100 mV to +60 mV in 20 mV steps. Recordings were performed using an internal solution containing (in mM) 110 NaF, 20 NaCl, 10 EGTA, and 10 HEPES, pH 7.2 adjusted with KOH. The bars on top show the duration of illumination. (C) Current-voltage relationships of NCR1 2.0 and XXM 2.0 are shown as mean ± SEM. (D) The reversal potentials of the current at the end of illumination are shown for both channelrhodopsins. (E) The shifts in reversal potentials following replacement of intracellular K^+ to Na^+. The bars are mean ± SEM, the dots are data points from individual cells. Statistical significance was assessed using an unpaired t-test. NCR1 2.0: n = 5(17); XXM 2.0: n = 9(22); ** < 0.01; * < 0.0001. n represents the number of responding cells for a given experimental condition out of the total amount of valid cells given in brackets.*

Table S1: *The table shows the Min. pulse width, the Min.-Max. LED current, the Max frequency, the Max. Light output noise and the Min. Resolution parameters for SOL as specified by Nanion Technologies GmbH.*

Min. pulse width	Min.-Max. LED current	Max. frequency	Max. Light output noise	Min. Resolution
0.8 ms	0.5 mA - 900 mA	100 Hz	<1% @100 mA<br><2% @200 mA	200 µA
